# Prevention of War: A Scoping Review on Primary Preventive Measures in Public Health

**DOI:** 10.3389/phrs.2023.1606201

**Published:** 2024-01-04

**Authors:** Tessa-Maria Brake, Oliver Razum

**Affiliations:** Department of Epidemiology and International Public Health, School of Public Health, Bielefeld University, Bielefeld, Germany

**Keywords:** public health, war, scoping review, primary prevention, armed conflict

## Abstract

**Objectives:** Wars and armed conflicts have a major impact on population health. As the discipline of public health aims to increase the health at population level, professionals play a significant role in dealing with war and armed conflict. There is need for research on prevention. This study aims to map the literature on existing public health approaches addressing the primary prevention of war and/or armed conflict.

**Methods:** We performed a scoping review in the databases Web of Science, PubMed and Google Scholar, followed by a narrative synthesis.

**Results:** We included 15 studies. We identified three main themes regarding preventive measures: 1) *research* on root causes of conflicts, surveillance and documentation of its health consequences; 2) *education* and awareness raising on the consequences of conflicts; and 3) *interventions* to change socio-economic and political conditions conducive to conflicts.

**Conclusion:** A two-tiered conceptual framework emerges: For primary prevention of war, public health should promote human rights and the rule of law. To prevent armed conflict within states, public health should address the social determinants of health and aim to reduce poverty and inequity.

## Introduction

War is commonly defined as “a state of usually open and declared armed hostile conflict between states or nations” [1]. However, this definition is no longer adequate because it does not consider the most common types of armed conflict: Since 1945, few wars have involved the annexation of an entire country or large parts of a country [2]. Indeed, “we are increasingly facing internal armed conflicts, wars within states, and often conflicts involving non-state actors such as private armies and locally armed militia.” [3, p.678]; but also conflicts with at least one state actor [3]. International humanitarian law distinguishes between two types of armed conflict: Firstly, international armed conflicts, which are fought between two or more states, and secondly, non-international armed conflicts, which are fought between state armed forces and non-state armed groups or only between such groups [4]. Recent examples for the latter are the armed conflict in Sudan which began in April, 2023; and Hamas’s attack on Israel on 7 Oct 2023, followed by the war in Gaza. However, Russia’s war of annexation in Ukraine or China’s renewed threat to invade Taiwan show that wars of aggression between states are still relevant. To cover the entire range of definitions in this scoping review, we use both the term *war* and the term *armed conflict*. War is more oriented towards war between states, armed conflict towards conflicts within states.

Wars and armed conflicts have a major impact on the health of populations, making them a public health issue. Direct consequences include increased mortality and morbidity among civilians and combatants [5]. Indirect consequences are more difficult to assess. They include psychological trauma, increased need for medical treatment and rehabilitation of war victims, impoverishment of disputed areas, and a collapse of institutions, such as in health, security, and education [5]. Wars and armed conflicts also impair the environment, economies, and food security in the areas directly affected, or even globally, as the war in Ukraine is showing. These indirect consequences often cause more deaths in the long run than the war/conflict itself [6, 7].

Ensuring that all people live in peace and prosperity by 2030 is the main goal of the Sustainable Development Goals (SDGs). SDG16 in particular stands for reducing all forms of violence and related death rates [8]. As the discipline of public health aims to improve the health at population level, for example, through health interventions, public policy and education, it plays a significant role in dealing with the consequences of wars and armed conflicts. The only way to completely avoid these consequences, and thus safeguard the health of the population, is through prevention strategies. However, these pose a great challenge [9]. To analyze the potential stages at which war/armed conflict may be prevented or its effects minimized, the model of primary, secondary, and tertiary prevention—originally from the field of medicine—is often used. Primary prevention aims to prevent an event before it occurs and includes, among others, the creation of a healthy and safe environment, avoidance of health-damaging behaviors, and the promotion of associated skills, knowledge and understanding. Secondary prevention is about stopping events at an early stage, usually through early detection—in the case of conflicts at an initial stage. Tertiary prevention aims to reduce disability or death when conflicts are already underway [10]. Ideally, war should be prevented altogether by focusing on primary prevention strategies. Research in this area is urgently needed [11–13].

This review aims to map the literature on existing public health approaches addressing the primary prevention of war and armed conflict in order to provide an overview of the topic and identify key gaps, including suggestions for future research needs.

## Methods

We conducted a scoping review to examine the current state of empirical research on public health frameworks, guides, or approaches addressing the primary prevention of war and armed conflict. A scoping review aims to capture the range of evidence in a particular research area, identify potential gaps and synthesize knowledge [14]. Unlike a systematic review, it allows for a broader scope and iterative approaches that are useful for identifying the nature and extent of ongoing research in all formats, including grey literature [14], in line with our study objectives. We followed the steps of the PRISMA-ScR protocol [15], thereby ensuring transparency and reproducibility.

### Data Sources and Search Strategy

We searched PubMed, Web of Science and Google Scholar—considered as a database for grey literature—for literature published in English and German and available in full text; no restrictions were placed on the date and type of publication. We also searched the reference lists of the papers for further relevant literature. The search string included the principal terms “war,” “armed conflict,” “Public Health,” “Public Health framework” and “interven*,” “prevent*,” “measure*,” “action*.” The search was performed in February 2023 using the following search string in PubMed:

(war[Title/Abstract] OR “armed conflict”[Title/Abstract]) AND (“Public Health”[Title/Abstract] OR “Public Health approach*”[Title/Abstract] OR “Public Health framework*”[Title/Abstract] OR “Public Health guid*”[Title/Abstract]) AND (interven*[All Fields] OR prevent*[All Fields] OR measure*[All Fields] OR action*[All Fields]))

The search string was adapted to the other databases accordingly.

### Study Selection

We screened titles and abstracts of records against a set of pre-defined inclusion and exclusion criteria. To cover a wide range of literature and existing definitions (see introduction), we use the terms *war* and *armed conflict*. To be eligible, records had to include the search terms “war,” “armed conflict” or any of the terms related to public health contained in the search string in their title or abstract. References had to describe public health approaches, frameworks or guidance reporting on primary prevention measures, actions or recommendations regarding war and/or armed conflict. We aim to provide an overview of the existing literature, so we posed no restrictions regarding the type of article (empirical, conceptual, etc.). Records focusing on consequences, outcomes, or impact of war, armed conflict, or (bio)terrorism on population health were excluded. We retrieved full texts of potentially eligible references and re-assessed them against the inclusion and exclusion criteria (see Table 1).

**TABLE 1:** Inclusion and exclusion criteria (Prevention of war: A scoping review on primary preventive measures in public health, Bielefeld, Germany. 2023).

Inclusion	Exclusion
Search term components “war” and “public health” and “framework” as well as related terms (see search string in chapter “data sources and search strategy”) are mentioned in Title/Abstract	Studies with focus on
	• Effects/consequences/outcomes/impact of war/armed conflict on population health
	• Nuclear/chemical weapons/war, (bio)terrorism
	• Violent conflict in general (sexual, intimate partner, etc. violence)
	• War on/prevention/epidemiology of COVID-19 and other diseases
	Studies not including a framework/approach, etc. of/regarding public health
	Studies including approaches to other public health topics than war/armed conflict
	Studies on war as a factor for other kinds of violence (intimate partner, sexual, etc.)
(Clear/direct) connection to war/armed conflict is made	See above
Includes role of public health (profession)	No mentioning of role of public health (profession)
Written in English or German	Written in other languages

### Data Extraction and Analysis

We extracted data using a template covering features of primary prevention strategies regarding war and/or armed conflict. In addition, we documented challenges and limitations raised in the references to provide suggestions for further research. We followed the principles of narrative synthesis based on the Guidance on the Conduct of Narrative Synthesis in Systematic Reviews which is the most appropriate approach for analyzing diverse evidence [16]. Scoping reviews do not require a formal quality assessment; which in addition would have been challenging due to the many different types of papers included. This scoping review did not involve primary research with human subjects and therefore did not require institutional ethical approval.

## Results

**FIGURE 1:**
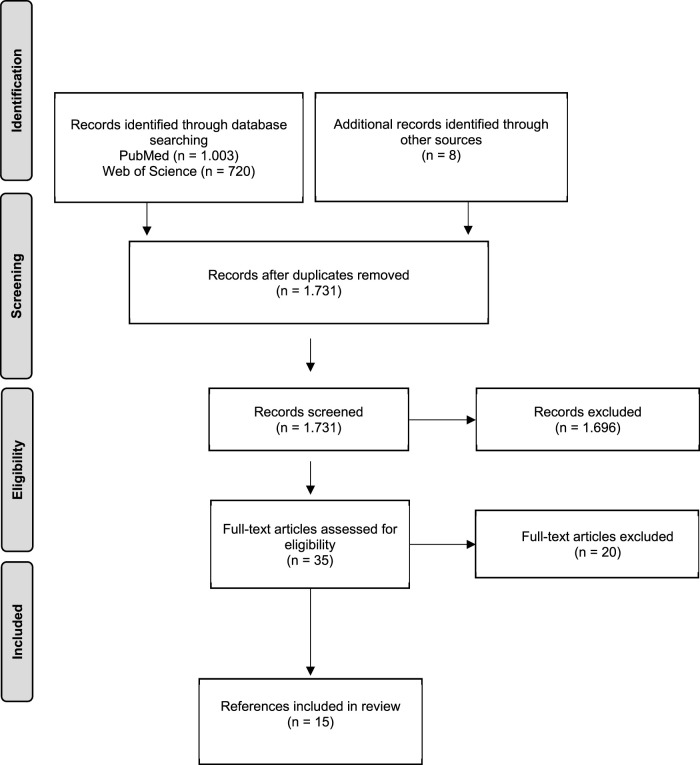
Flow chart depicting literature search and exclusion process (modified according to The Joanna Briggs Institute Reviewers’ Manual [17]) (Prevention of war: A scoping review on primary preventive measures in public health, Bielefeld, Germany. 2023).

We identified a total of 1,723 records following the removal of duplicates and eight additional records in our Google Scholar search. After an initial screening of titles and abstracts, we considered 34 references for full text review. Of these, we identified 15 as eligible for inclusion (see Figure 1). An overview of the studies can be found in Table 2.

**TABLE 2:** Characteristics of studies included (Prevention of war: A scoping review on primary preventive measures in public health, Bielefeld, Germany. 2023).

Reference	Type of article	Outcome
APHA (2009) [18]	Policy statement	• *Research on the root causes* of war (on-going because causes and patterns of war change) and surveillance (estimates of deaths to raise awareness)
		• *Education* of public health professionals, policymakers, and the public about the expected consequences of war and to advocate for alternative solutions to conflict
		• *Change in conditions*: call for an updating and strengthening of the role of public health professionals
Burkle (2019) [19]	Framework	• Interdisciplinary collaboration *in research and surveillance*, particularly between natural and social sciences, such as in global health
De Jong (2010) [20]	Framework (based on a selective literature review)	• Interdisciplinary collaboration *in research and surveillance*: risk and protective factors are poorly confirmed by research; little is known about the extent to which these factors operate alone or in combination with other factors
		• *Education* of public health professionals, policymakers, and the public about the expected consequences of war and to advocate for alternative solutions to conflict; health, education, and other sectors could promote reconciliation and cooperation by, e.g., setting policies to strengthen equitable health and education services, developing human resources through a cascade of training levels, providing educational materials, and establishing a monitoring and supervision system
		• *Change of conditions*: need for effective police authority and fair criminal and dispute resolution systems; international law should focus on three areas: human rights, humanitarian laws and non-violent alternatives to dispute resolution; reducing unemployment and poverty, protecting the environment and ensuring basic security, wellbeing and justice; reduction of discrimination and ethnic inequalities; remove barriers to equal opportunities by providing equal access to basic facilities/services such as health services and education
Grundy et al. (2008) [21]	Conceptual framework	• Early warning and *surveillance* systems
Hagopian and Samer (2022) [22]	Statement	• *Education and awareness*: public health professionals need to address and be encouraged to address the prevention of war and to build a movement for peace
		• *Change of conditions*: important to consider the aspects of corruption and greed in the war industry
Lang et al. (2002) [23]	Strategy paper	• *Education and promotion* of tolerance
		• *Change of conditions:* states should be disarmed or at least be reduced to a minimum of weapons, with a strong international capacity to mediate and intervene
Levy and Sidel (2003) [24]	Discussion and analysis paper	• Promoting a culture of peace through *education*
		• *Change of conditions:* addressing social, economic and cultural factors leading to war: poverty, social injustice; strict control of weapons
Levy et al. (2017) [25]	Approach	• Need on valuable *research*: risk and protective factors are poorly confirmed by research; little is known about the extent to which these factors operate alone or in combination with other factors
		• *Raising awareness* of consequences of war among professionals, NGOs, policymakers and public
		• *Change of conditions:* working towards reducing poverty, income inequality, ethnic hatred; focus on climate change
Levy, B. (2022) [26]	Book chapter	• Need for further *research* on risk and protective factors and their interaction
		• *Education and awareness raising:* most important: dialogue and diplomacy: can prevent disputes and conflicts from arising through various forms of dialogue such as face-to-face negotiations and conferences
		• *Change of conditions:* poor governance can contribute to the emergence of wars: improving citizen participation, e.g., through free elections, can make the government more accountable and strengthens governance
Murray et al. (2002) [27]	Approach	• Need in *research*: collaboration between political scientists and public health researchers
Reza et al. (2018) [28]	Framework	• Need for further *research* on risk and protective factors and their interaction; identification of risk and protective factors: public health professionals should develop and assess interventions for the primary prevention of armed conflict and its health consequences, strategies to be evaluated: economic sanctions, peacekeeping measures by the United Nations, education about the danger of landmines as preparation of the civilian population for a possible war; identification of the most effective methods (together with epidemiologists and professionals in other relevant scientific disciplines)
		• *Education* of the public and professionals
Sidel and Levy (2006) [29]	Commentary	• Argue for a *surveillance* system to monitor health outcomes
Valenti et al. (2007) [5]	Approach	• Identification of risk factors and scope of problem through data collection/*research*
		• Education of decision-makers about health consequences and human costs; raising awareness through public health campaigns (e.g., mass media campaigns); advocacy of (more) comprehensive health and medical policies addressing root causes of war
Wiist et al. (2014) [30]	Commentary	• *Research and surveillance*: conducting regular risk and policy analyses and environmental assessments, especially those involving multiple stakeholders
		• *Education and awareness raising*: inclusion of war and armed conflict in school curricula
		• *Change of conditions*: (stronger) control, reduction and outlawing of weapons within states, e.g., through appropriate legislation, or multilateral or bilateral treaties; updating and strengthening of the role of public health professionals (focus too much on the effects of war rather than on its prevention)
WHO (2020) [12]	Approach	• *Research*: need to conduct and/or learn from existing conflict/risk analyses, inclusion of stakeholder analyses and mapping root and proximate causes, triggers, conflict dynamics and peace capacities, identification of relevant drivers
		• *Education/awareness raising:* Suggests the design of peace-responsive health interventions: aim to improve trust between potential groups of conflict 1) improve trust between the state and its citizens by, e.g., expanding social protection and justice in underserved areas; 2) public health professionals could serve as a platform for belligerents restoring contacts and collaborations 3) need of trust-building and inclusive processes that promote dialogue to improve the relations between individuals and communities

The type of references are statements, commentaries or frameworks/approaches and one book chapter. Seven of them were published before 2010, the other eight after 2010. The references are clustered according to three main themes regarding primary preventive measures: 1) *research* on root causes of conflicts, surveillance and documentation of health consequences, 2) *education* and awareness raising on the consequences of conflicts and promotion of peace, and 3) *interventions* to change socio-economic and political conditions conducive to conflicts.

### Research on Root Causes of Conflicts, Surveillance and Documentation of Health Consequences

Twelve of the 15 references [5, 12, 18–21, 25–30] mentioned the aspect of examining the root causes of war and/or measuring health consequences as first basic actions to prevent war and armed conflict.

According to the American Public Health Association [18], continuous research into the causes of wars/armed conflicts is essential to prevent them. They argue that wars and armed conflicts need to be investigated not only when they break out, but already before. This would require a refinement and more effective design of public health tools to produce pre-conflict estimates on, for example, deaths to raise awareness and ongoing surveillance during conflicts regarding the impact on health systems [18]. Burkle [19] and De Jong [20] support these approaches, but focus on interdisciplinary collaboration in research and surveillance, particularly between natural and social sciences, such as in global health. The problem, according to Burkle [19], is that it is currently unclear to what extent such collaboration is actually taking place. Murray et al. [27] go into more detail here, emphasizing the importance of collaboration between political scientists and public health researchers. In addition, Sidel and Levy [29] argue for a surveillance system to monitor health outcomes. Another approach also proposes the development of such a system but with a focus on risk factors of war and armed conflict [28]. At the same time, the risk and protective factors of war and armed conflict are poorly confirmed by research, and little is known about the extent to which these factors operate alone or in combination with other factors [20, 25, 28]. Building on this, it is relevant for the primary prevention of war to conduct regular risk and policy analyses and environmental assessments, especially those involving multiple stakeholders [12, 30].

Building on the identification of risk and protective factors, public health professionals should also develop and assess interventions for the primary prevention of armed conflict and its health consequences, according to Reza et al. [28]. As strategies to be evaluated, they list, for example, economic sanctions, peacekeeping measures by the United Nations or education about the danger of landmines as preparation of the civilian population for a possible war [28].

### Education (of Professionals, Policymakers and the Public) and Awareness Raising on the Consequences of Conflicts and Promotion of Peace

11/15 references [5, 12, 18, 20, 22–26, 28, 30] mentioned educating professionals and the public as well as raising awareness on the consequences of war and promoting peace as further fundamental public health approaches to the primary prevention of war and armed conflict.

According to APHA (American Public Health Association) [18] and De Jong [20], it is relevant to educate public health professionals, policymakers, and the public about the expected consequences of war and to advocate for alternative solutions to conflict. They, as well as Hagopian and Jabbour [22], argue that it is necessary for professionals to address and be encouraged to address the prevention of war and to build a movement for peace. APHA [18] recommends that public health professionals should advocate for legislation related to the arms trade, ratification of treaties and military spending, financial and political engagement in peace operations, and development programs that address the structural causes of war. Professionals should also address the public health impact of war [18]. To achieve this, the (primary) prevention of war and armed conflict should be included in school curricula [18, 30]. Particular important would be the promotion of the peace-making function of public health professionals [18]. De Jong [20] supports these approaches. He argues that access to health and education services is often limited due to various factors, and military action often undermines public health programs. Therefore, he points out that health, education, and other sectors could promote reconciliation and cooperation by, for example, setting policies to strengthen equitable health and education services, developing human resources through a cascade of training levels, providing educational materials, and establishing a monitoring and supervision system [20]. Wiist et al. [30] mention necessary skills of Public Health professionals regarding trust- and peacebuilding. Thus, professionals could bring parties together to cooperate and coordinate health activities. The World Health Organization (WHO) [12] supports this approach and suggests the design of peace-responsive health interventions with the aim to improve trust between potential groups of conflict. First, they point out that it would be important to improve the trust between the state and its citizens by, for example, expanding social protection and justice in underserved areas. Second, public health professionals could serve as a platform for state belligerents restoring contacts and collaborations [12]. Finally, trust-building and inclusive processes that promote dialogue are needed to improve the relations between individuals and communities; health dialogue and diplomacy are considered particularly important [12]. Levy [26] supports the approach of dialogue and diplomacy, describing the latter one of the most important approaches to preventing wars, as it can prevent disputes and conflicts from arising through various forms of dialogue such as face-to-face negotiations and conferences.

### Interventions to Change Socio-Economic and Political Conditions

A higher-level public health issue that we found in eight out of 15 [18, 20, 22–26, 30] of the references was the primary prevention at the policy and administrative level.

Five Refs. [20, 23, 24, 26, 30] refer to (stronger) control, reduction and outlawing of weapons within states, for example, through appropriate legislation, or multilateral or bilateral treaties. Lang et al. [23] advocate that states should be disarmed or at least be reduced to a minimum of weapons, with a strong international capacity to mediate and intervene. A framework based on De Jong [20] emphasizes the need for effective police authority and fair criminal and dispute resolution systems. Furthermore, international law should focus on three areas: human rights, humanitarian laws and non-violent alternatives to dispute resolution. Humanitarian laws include the need to legally underpin United Nations field operations and should also take into account the needs of vulnerable groups, religious freedom and the right to preserve non-harmful cultural practices [20, 25].

Beyond legislation, more fundamental initiatives such as rural development, for example, increasing food production, can help to strengthen economic capacity and improve food security, resilience and quality of life [20]. Particularly in areas of increasing instability, such projects can compensate for the lack of land and prevent competition between the local populations and internally displaced people or refugees [20]. This framework, based on a selective literature review that translates risk factors into preventive interventions at multiple levels, also suggests reducing unemployment and poverty, protecting the environment and ensuring basic security, wellbeing and justice [20]. In addition, discrimination and ethnic inequalities must be reduced and societies rebuilt [20]. States should remove barriers to equal opportunities by providing equal access to basic facilities/services such as health services and education [20]. Leaders of poverty-stricken countries, for example, that have abundant oil, minerals and other resources can use these resources to fight extreme poverty and socio-economic inequities [26]. Poor governance can also contribute to the emergence of wars. Improving citizen participation, for example, through free elections, can make the government more accountable and strengthens governance [26]. Levy et al. [25] also highlight the work to reduce poverty, income inequality and ethnic hatred, and also mention other causes that need to be addressed such as climate change and environmental degradation. Another approach identifies strategies related to governance and economics, geography, level of development, cultural factors and individual incentives. Finally, Hagopian and Samer [22] point out that it is important to consider the aspects of corruption and greed in the war industry. The World Health Organization [12] suggests—in their report on the work *on* conflict instead of only *in* conflict—to focus on those drivers that can be adequately addressed through interventions and are linked to health concerns.

The approaches identified also raise criticism. APHA [18] and Wiist et al. [30] argue that the public health profession avoids activities that could prevent wars because they are too controversial or political. Accordingly, they call for an updating and strengthening of the role of public health professionals [18, 30]. So far, these have focused on the effects of war rather than on its prevention [30]. Grundy et al. [21] argue that the decision to go to war is generally made regardless of the threat to public health. They criticize that public health remains on the margins of conflict awareness, decision-making, and mitigation, while political, technocratic, legal, and military advocates play the primary role [21].

Additionally, the references often do not directly distinguish between war and armed conflict in their approaches; the terms are mostly used synonymously. However, based on our definitions of these terms (see background chapter), primary prevention approaches can be derived for both wars of aggression and armed conflicts.

## Discussion

The aim of this study was to map the literature on existing public health approaches addressing the primary prevention of war and/or armed conflict. We identified a range of measures covering the areas of 1) *research* on root causes of conflicts, surveillance and documentation of health consequences, 2) *education* of professionals, policymakers and the public, awareness raising on the consequences of conflicts and promotion of peace, and 3) *interventions* to change socio-economic and political conditions.

A first striking finding was that many references focus on the prevention of armed conflict rather than on war of aggression, which include wars of annexation such as Russia’s war in Ukraine. One reason for this could be that since 1945, there have been few wars in which an entire country or large parts of it were annexed, and most studies included here were published before Russia’s full-scale invasion of Ukraine in 2022. Moreover, many references come from the same group of authors with a pronounced pacifist stance, who seemed to have been optimistic that such wars were no longer a major risk (especially Barry S. Levy and Victor Sidel). Ultimately, many papers remained on the surface of primary prevention measures.

The call for stricter gun laws and caps on military spending to prevent wars and armed conflicts may also stem from this pacifist orientation. Restricting gun ownership among civilians would likely prevent deaths and injury [20, 23, 24, 30]. Razum and Wandschneider [31] argue in their commentary, referring to Russia’s war in Ukraine, that public health professionals may have to concede that defense systems capable of balancing asymmetries in military power are needed to protect populations and their health from wars of aggression. They argue that it is therefore realistic for democratic nations to consider a well-equipped military as a means of primary war prevention.

Poor governance as one of the root causes of war is also a recurring theme. As a preventive strategy, for example, Levy [26] suggests that strengthening citizen participation, such as through free elections, can make the government more accountable. He argues that such mechanisms ensure justice and are thus essential to good governance. However, elections in countries whose current leaders conduct war or threaten to do so, such as Russia, North Korea, or China, can be considered neither free nor fair, based on country reports of human rights practices [32]. This raises the question whether Russia’s war of aggression in Ukraine, for example, could indeed have been prevented in this way. To answer this question, the motives and background of this war and also of other wars of aggression would have to be examined in more detail.

While strengthening human rights may well serve to prevent war, we found that public health professionals are mentioned mainly in roles in research as well as education and awareness raising among policymakers and the public, rather than in changing socioeconomic and policy conditions. In addition, most papers suggest few concrete measures to prevent war and armed conflict and only partially address public health. Due to criticism that wars are too controversial and political for the public health profession, there is a call to strengthen the role of the profession [18, 30]. Levy [26] and the WHO [12] argue for dialogue and diplomacy as one of the most important approaches to preventing wars. Many public health institutions, such as the Association of Schools of Public Health in the European Region (ASPHER) [33] or the Turkish Medical Association (TMA) [34], already position themselves as an important part of the prevention of war. However, Razum and Wandschneider [31] point out that activists against the war take high personal risks. TMA members, for example, were sentenced to prison after their protests and released only after international expressions of solidarity [35]. Razum and Wandschneider [31] advocate documenting health consequences, highlighting prevention options, and identifying and dismantling “othering” processes in and between societies to prevent division and hatred. These points are supported by the findings of this review.

Namer et al. [36] and Cunningham and Wandschneider [37] refer to taking a more active role in relation to schools of public health. Namer et al. [36] point out that it is important to broaden the scope to reflect how public health actors from many disciplines contribute to peacebuilding. Secondly, they state, that the schools are strongly committed to protecting the health of the population in the broadest sense, both in practice and in theory, by bringing together different levels and sectors. And third, Namer et al. [36] argue that schools of public health could use their tools to study, for example, the consequences of armed conflict on mental and physical health. Results of our review also highlight the relevance of public health schools and related training of professionals [18, 30]. However, public health schools currently lack appropriate curricula. According to White et al. [38], who examined the curricula of the top 20 public health schools (based on U.S. News and World Report’s 2011 program rankings) for primary war prevention content, only 0.5% (31/6,266) of the courses specifically addressed war and/or armed conflict. As of December 2023, ASPHER is starting to develop such a curriculum.

Public health professionals can help raise awareness about conflict prevention. This can include collecting accurate data, educating decision-makers and colleagues about the human costs of armed conflict, and advocating for more comprehensive health and medical policies [5]. Thus, new or amended laws can be passed or awareness raised (at national and international level), which in turn can have a preventive effect. At community level, public health professionals can take over the education of the public. In this way, a large number of people can be provided with information about help, legal rights and a variety of topics to help them cope with their particular situation [20]. An example of successful work by public health professionals is the International Physicians for the Prevention of Nuclear War (IPPNW), an international medical organization with the goal of preventing nuclear war and abolishing all nuclear weapons [39]. IPPNW conducts research on the impact of war on health, educates health professionals and other stakeholders, collaborates with other institutions and advocates for health-promoting policies [40]. This is largely consistent with our findings and suggests that these measures can be successful.

Public health professionals cannot act alone in preventing war and armed conflict. Multiple actors from different disciplines are required, particularly with regard to the structures and systems identified under the theme *policy and governance* that need to be fundamentally changed. For example, a holistic approach to sustainable food systems development, as called for in one approach in the review [20], is relevant because the challenges involved are multidimensional and interconnected [41]. This is again supported by the approaches identified in this review, which only partially address public health; they require a whole-of-society approach.

The evidence base for the effectiveness of current interventions is thin. This may be due to the complex interconnectedness and diversity of causes of war, which complicate the evaluation of interventions [42]. Although mentioned by only one source in our review, the assessment of such strategies is of great importance to ensure the sustainability and effectiveness of health interventions; it can reveal strengths and weaknesses and thus improve existing strategies [43]. One reason regarding the low number of mentions or implementation could be the paucity of research and the difficulty in conducting such evaluations. In addition, Cramer et al. [44] address two challenges in the context of war prevention: 1) long causal chains from interventions to impacts, and 2) fundamental challenges due to the lack of clear knowledge about the counterfactual situation. Furthermore, Böck Buhmann [42] points to the difficulty of finding methods for assessing the peace policy impact of an intervention and to methods that are applied so differently that comparison is problematic. Finally, from a statistical point of view, the number of wars is small, further complicating effectiveness measurements.

### Strengths and Limitations

By conducting a scoping review, we were able to examine a broad range of themes and identify gaps in the evidence of primary preventive measures regarding wars and armed conflicts in public health. Our search was extensive and based on defined inclusion and exclusion criteria. Upcoming issues were resolved in consultation with the co-authors. However, several limitations must be mentioned. Firstly, the primary search in the databases was conducted by a single reviewer, which may have led to bias regarding the decision (not) to include literature. To minimize this bias, a second reviewer checked the results and suggested additional references. The inclusion and exclusion criteria are very broad to be able to include as many studies as possible. This may make it difficult for other researchers to evaluate the findings. Another limitation is the lacking differentiation of the definitions of war and armed conflict due to the high heterogeneity of the use of these terms in the present studies. This has made our analysis very broad. There may also have been a bias in identifying and documenting findings due to the restriction to German and English articles.

### Conclusion and Recommendations

The aim of this review was to survey the literature on existing public health approaches to primary prevention of war and armed conflict to provide an overview of the topic and identify key gaps, including suggestions for future research needs. Notable were the focus on armed conflict rather than on wars of aggression; and the small number of concrete measures addressing the primary prevention of war from a public health perspective.

The results can be condensed in a two-tiered conceptual framework:

To prevent wars between states, public health can promote human rights and the rule of law, either directly through political work or indirectly through equitable access to justice and interventions against discrimination or gender-based violence.

To prevent armed conflicts within states, public health interventions should address the social determinants of health and aim to reduce poverty and inequality. This can be achieved by improving access to education and healthcare. Education can promote understanding and dialogue between groups. Such interventions can strengthen social cohesion and help reduce the likelihood of conflict and violence. Economic development also plays a role, but falls outside the scope of public health (apart from the fact that health is a prerequisite for participation in the labor market). Again, public health can promote human rights and the rule of law, either directly through policy work or indirectly through equitable access to justice and interventions against discrimination or gender-based violence.

Further research should focus not only on armed conflicts but also on wars of aggression. Researching the causes of armed conflicts and wars as well as their influence on each other is a prerequisite for developing preventive measures and testing their effectiveness. Since wars and armed conflicts are often associated with poverty or social inequality, a holistic society approach is needed for their primary prevention.

## Abbreviations

APHA, American Public Health Association; ASPHER, Association of Schools of Public Health in the European Region; IPPNW, International Physicians for the Prevention of Nuclear War; SDGs, Sustainable Development Goals; TMA, Turkish Medical Association; WHO, World Health Organization.
